# Novel and Reusable Graphene Oxide-Coated Reticulated Open-Cell Mullite Foams for Methylene Blue Dye Adsorption

**DOI:** 10.1021/acsomega.3c07569

**Published:** 2024-01-24

**Authors:** Wadwan Singhapong, Angkhana Jaroenworaluck, Panlekha Manpetch

**Affiliations:** National Metal and Materials Technology Center (MTEC), National Science and Technology Development Agency (NSTDA), 111 Thailand Science Park, Phahonyothin Road, Khlong Nueng, Khlong Luang, Pathum Thani 12120, Thailand

## Abstract

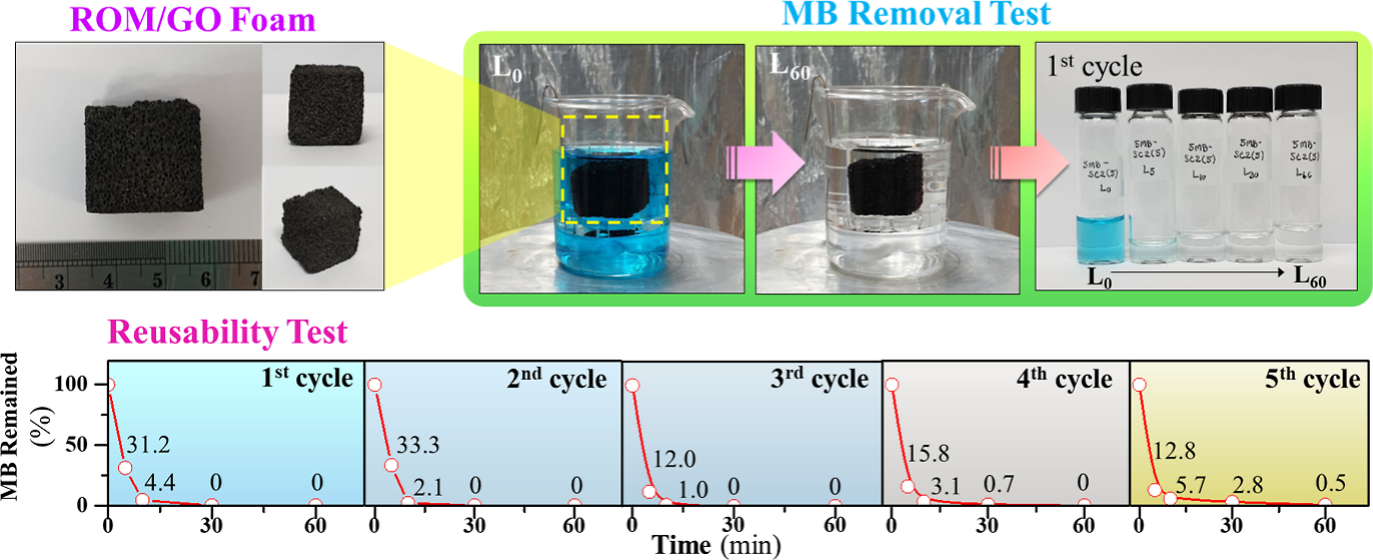

Reticulated open-cell mullite (ROM) foams coated with graphene oxide (GO) multilayers as novel and reusable composites (ROM/GO) were first fabricated and functionalized to remove methylene blue (MB) dye from aqueous solutions. In this study, the ROM foams were produced via a replica technique utilizing rice husk as a starting raw material of silica (SiO_2_) mixed with commercially available alumina (Al_2_O_3_) in the step of slurry preparation. GO was synthesized by a modification of Hummer’s method and dispersed in a fixed weight ratio in an aqueous solution. The HCl-pretreated ROM foams were dip-coated for up to 5 cycles using a fixed weight ratio of GO in an aqueous solution. The experimental results revealed that the ROM/GO foams provided 100% adsorption efficiency for MB within 30 min. The adsorption kinetics of the ROM/GO foams followed the first-order model. Based on the microstructural investigations on the surfaces of the ROM foams compared with the ROM/GO foams, the adsorption performance was related to the unique porous structure of the ROM foams, incorporating the physicochemical properties of the GO-multilayered coating. Finally, the ROM/GO foams are considered as sustainable and cost-effective adsorbents due to their reusable functionality for at least 5 cycles of MB removal.

## 1 Introduction

As clean water resources in the world are increasingly limited, technology development for the removal of contaminants from wastewater has been a severe challenge for decades. Costs of the technology, relating to maintenance costs, high concentrations of pollutants in wastewater, and the amount of wastewater to be treated are considered as the key factors in selecting the technology applied for wastewater treatment in practical terms. When compared with other wastewater technologies, adsorption is a more beneficial technology than the others due to its low costs and managed simplicity.^1−4^ However, costs of adsorbents and how to collect or recycle the used adsorbents are still big challenges to overcome these problems.^2−5^

Graphene oxide (GO), an advanced carbon material, is an atomically two-dimensional (2D) carbon arrangement. GO is generated from the oxidation of graphite.^6,7^ Due to the surface of GO containing functional groups of hydroxy, GO is a hydrophilic material and has the potential to adsorb contaminants such as Pb^2+^,^6,8^ F^+^,^9^ and heavy metal ions in wastewater.^3,6,9,10^ This allows GO to be used as an effective adsorbent to purify water and wastewater.^6,11,12^ To increase the GO efficiency, GO can be composited with other absorbents or support materials as hybrid-composites to promote the total removal performance for treating the targeted pollutants.^3,6,13^ However, coating GO layers onto the surfaces of support materials including porous materials can be an alternative.

It has been well-known that porous ceramics have been used in various kinds of industries due to their pore structure, lightweight, high porosity relating to high surface areas, low thermal conductivity, etc.^14,15^ Porous ceramics, containing large pores, ranging from macropores to micropores, are of interest as membranes, filters, and catalyst-supports to catch contaminants in water and air systems.^16−19^ Open-cellular ceramic foams have been considered as one of the most fascinating porous ceramics and can be manufactured as commercial products since their fabrication processes are simple and have low costs compared with other porous ceramics.^17^

Reticulated open-cell ceramics, also called ceramic foams, having a three-dimensional (3D)-interconnected pore configuration, have been regarded as one of the most promising ceramic foams, used in a wide range of industrial applications such as molten metal filtration,^20^ lightweight construction materials,^14^ catalyst supports,^16,18,19^ heat exchangers for thermal energy storage,^21^ bone replacement materials,^22^ and radar/solar absorption materials.^15,23^ The excellent properties of ceramic foams enable the foams to be exploited in the field of water treatment.^6,17−19^

Mullite (3Al_2_O_3_·2SiO_2_), an alumino-silicate material, is a candidate and is favorable among the structural oxide ceramics due to its high mechanical strength, good thermal-chemical resistance, and low values of dielectric constants and dielectric loss properties.^24−26^ In terms of the economic benefit aspect, mullite can be synthesized from numerous low-cost materials from natural resources such as clay or alumino-silicate ores^27^ agricultural sources like rice husk (RH) or RH ash,^26,28^ and industrial wastes from coal fly ash or aluminum slag.^24,25^ Singhapong et al. showed that reticulated open-cell mullite (ROM) foams, utilizing RH as a starting raw material, were successfully fabricated and scaled-up for further use.^28^ However, the nature of ROM foams is stable and not active due to the sintering process. Coating is one of the techniques that can extend the functions of ROM foams. Based on our knowledge, coating GO on the surfaces of the ROM foams is a novel approach to improve the adsorption ability and to reduce the additional cost paid for the post-treatment to separate the used adsorbents from the treated wastewater. In particular, the foams have never been applied to the removal of methylene blue (MB), which is a cationic dye, commonly used in various industries and found in discharged wastewater. It has been known that MB can not only create adverse effects on the aquatic ecosystem, leading to a decrease in downstream beneficial uses but can also potentially have carcinogenic and mutagenic effects on human health.^29^ Research on developing GO using adsorbents used in the powder form has been obvious, but few research works have discussed coating GO onto substrates/materials to treat MB.^30−32^ GO has been effectively used for the adsorption of MB.^4^ However, most of the research works revealed that GO adsorbent was used in a powder form and cofunctioned with photocatalyst materials such as TiO_2_ and ZnO.^33^

In this study, ROM foams were fabricated and coated with GO multilayers using a fixed weight ratio of GO in an aqueous solution of GO to form a novel type of composite (ROM/GO) foams to investigate the removal efficiency of MB by the adsorption process. To verify the economic feasibility of the ROM/GO foam performance, a reusability test was performed. The foam microstructures, typically pore structures relating to the adsorption performance, were investigated, studied, and their relationship discussed in detail to increase the performance.

## 2 Experiments

### 2.1 Chemicals and Materials

All chemicals used in the synthesis of GO were analytical grade. H_2_SO_4_ (98% w/w) was from Carlo Erbo Co., Ltd., Italy. H_3_PO_4_ (85% w/w) was from RCI Labscan Co., Ltd., Thailand. HCl (37% w/w) and KMnO_4_ were obtained from Thermo Fisher Co., Ltd., Australia. H_2_O_2_ (>30% w/v) was from Fisher Scientific Co., Ltd., UK.. Alumina powder (Al_2_O_3_) donated by RiO Tinto Alcan Inc., Canada, and RH from a rice mill in Saraburi province, Thailand, were used as raw materials in the fabrication of the ROM foams.

### 2.2 Fabrication of the ROM Foams

The ROM foams were fabricated by a replica technique, as reported in the previous study.^28^ Briefly, in this study, the M4 composition reported in the previous study (Al_2_O_3_: pretreated RH = 72:28 by weight) with 1% of the total solid weight of activated carbon added was used as the starting raw material to prepare the slurry via ceramic processing. Polyurethane foams, having a 45 ppi pore size, were used as the pore templates of macropores. The polymer foams were cut into a size of 60 × 25 × 25 mm, coated with the prepared slurry, dried, and finally sintered at 1500 °C for 4 h. Prior to use, the sintered foams were cut into a size of 30 × 25 × 25 mm^3^ and pretreated with a 6 M HCl solution prior to the coating process to increase surface adhesion between the ROM foams and the GO layers.

### 2.3 Synthesis of the GO Powder

In this study, GO was synthesized via a modified Hummers method, using pencil leads as a source of graphite material, as reported in the previous studies.^33,34^ Briefly, 3.0 g of the prepared graphite powder was chemically oxidized using KMnO_4_ as the oxidizing agent in an acidic solution mixture of H_2_SO_4_ and H_3_PO_4_. The reaction was carried out at 50 °C with stirring for 36 h. Ice cubes made from deionized (DI) water and 30% H_2_O_2_ were then added to prevent further oxidation. The resulting solution was sonicated for 10 min followed by washing with 10% HCl and DI water to reach a neutral pH using centrifugation. The obtained GO powder was finally dried at 60 °C overnight.

### 2.4 Coating Process

Multilayers of GO were coated on the surfaces of the ROM foams. The synthesized GO was mixed with DI water as a GO solution (0.5 g/L). The ROM foams were carefully coated with the GO solution using dip coating and dried at 60 °C to complete the initial coating layer. After five cycles of the coating process were repeated, the ROM foam surfaces were fully covered by GO layers. The coated foams were designated as ROM/GO foams.

### 2.5 Adsorption and Reusability Tests

The adsorption performance of the ROM/GO forms was evaluated by using the MB solution as a model pollutant of dyes. The experiment was conducted in a batch test using a homemade apparatus. The initial concentration of MB was set at 5 ppm. The ROM/GO foams were immersed in the MB solution with magnetic stirring. Treated solutions with the ROM and ROM/GO foams were collected at different time intervals from 0 to 60 min. MB concentrations decreased in the solutions were evaluated using a UV–vis spectrophotometer (V-750, Jasco, Japan) at the λ_max_ of 664 nm. The reusability test was performed without any further chemical requirement by desorbing the used ROM/GO foams in DI water under stirring, sonicating, and subsequently drying before repeating the adsorption test. Dye removal was calculated according to eq 1
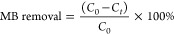
1where *C*_0_ and *C*_*t*_ are the initial concentration of MB (ppm) and the concentration of MB (ppm) at each time interval, respectively.

To investigate kinetic model studies, the pseudo-first-order model was used for evaluation as shown in eq 2
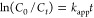
2where *C*_0_ is the initial concentration of MB (ppm), *C*_*t*_ is the concentration of MB (ppm) at each time, and *k*_app_ is the apparent reaction rate constant determined from slopes of the linear plots of −ln(*C*_*t*_/*C*_0_) in a function of reaction time.

### 2.6 Characterizations

The phase compositions of the ROM foams and the synthesized GO were identified by an XRD diffractometer (XRD; PANalytical X’Pert PRO, Netherlands). Flexural strength and flexural modulus of the ROM foams were carried out using a universal testing machine (Instron 5943, Instron, USA). Microstructures of the foams and the synthesized GO were observed by field emission scanning electron microscopy (FE-SEM; S-3400 and SU5000, Hitachi, Japan) and transmission electron microscopy (TEM; JEM 2010 plus, JEOL, Japan). The surface chemical compositions of the synthesized GO were investigated by X-ray photoelectron spectrometry (XPS; AXIS Supra, Kratos Analytical Ltd., Co., UK).

## 3 Results and Discussion

### 3.1 Surface Appearance of ROM/GO Foams

**Figure 1:**
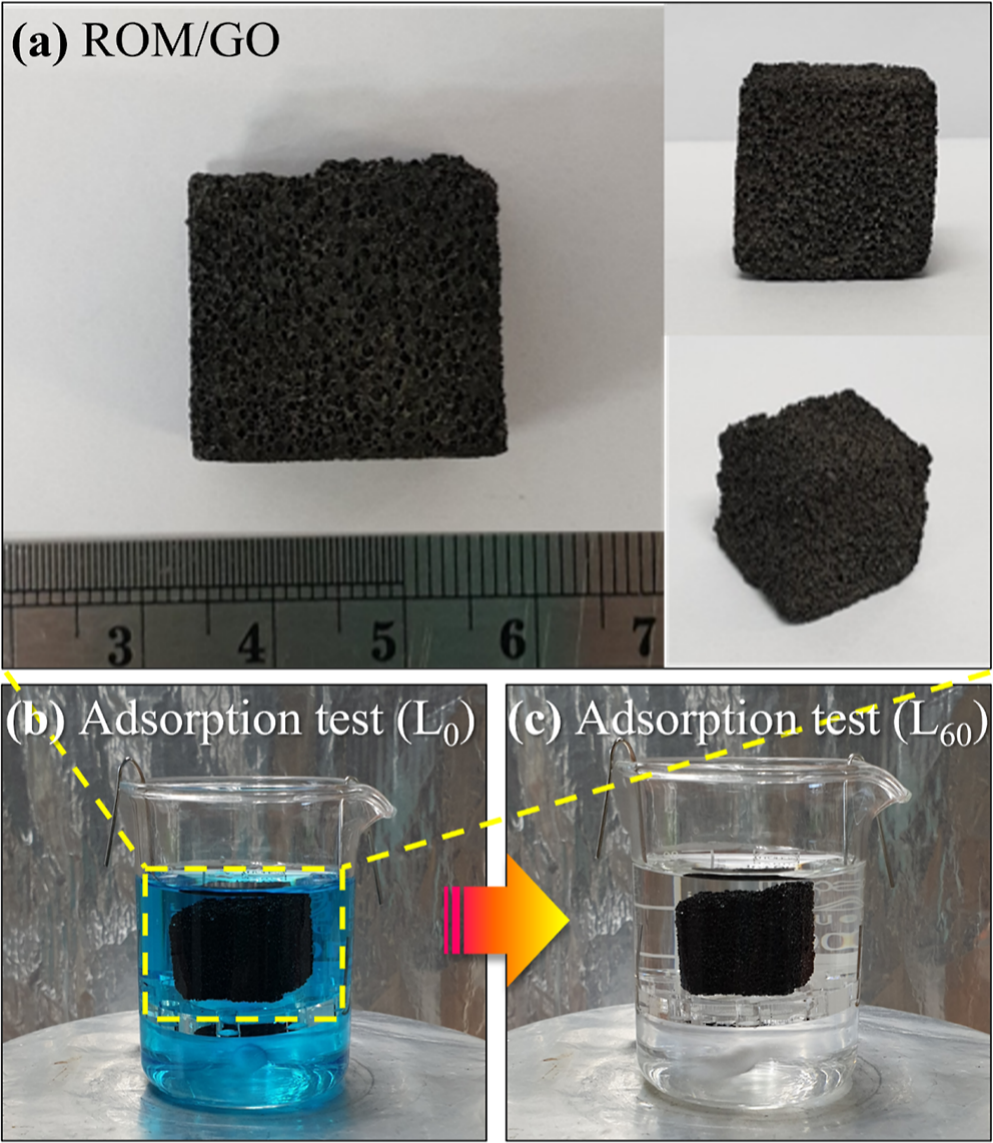
Photographs of (a) ROM/GO foam and (b,c) MB adsorption tests with a ROM/GO foam at 0 and 60 min, respectively.

Figure 1a shows the attachment of GO layers on the surface of a ROM foam, as indicated by the black appearance of the ROM/GO foam surface. During the coating process, it was obvious that the coating thickness of the GO layers relatively increased by an increase of the entire weight of the foams in each coating cycle, implying an effective method to coat GO onto the surfaces of the ROM foams. The strong adhesion of GO may be contributed to the development of surface roughness of the foam by the acid treatment, leading to hydrogen bonding between oxygen functional groups on the surface of GO and hydroxyl groups on the surface of the foam.^35^

### 3.2 MB Adsorption Performance

**Figure 2:**
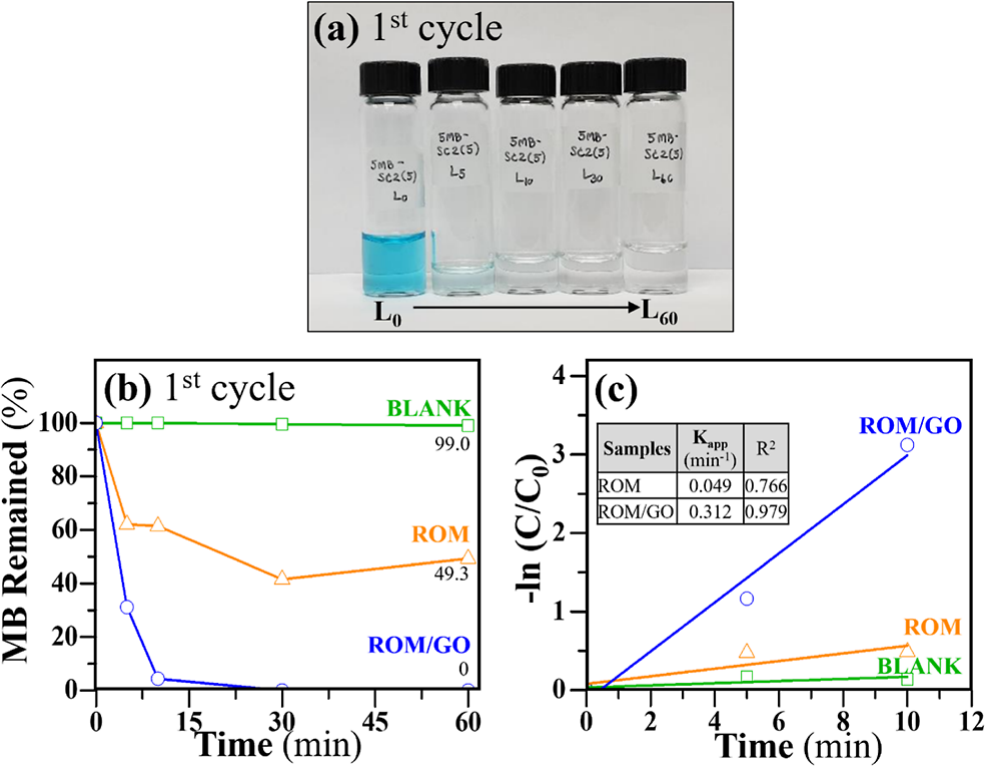
(a) Color changes of the MB solutions at different time intervals, (b) profiles of MB remaining in the solutions adsorbed by the ROM/GO foams compared with the ROM foams, and (c) pseudo-first-order kinetic plots for the MB adsorption.

The MB adsorption performance of each ROM/GO foam was set up in a batch test, as shown in Figure 1b. The MB solution was changed from blue to colorless after 60 min of adsorption, as clearly observed in Figure 1c. Figure 2a reveals the change in color of the MB solution during adsorption by using ROM/GO foam. Notably, the blue color of the MB solution gradually lightened and visibly disappeared within 10 min. The MB removal efficiencies of the ROM/GO foams compared to those of the ROM foams are shown in Figure 2b. Although the ROM foams could absorb 49.3% of MB after 60 min, the ROM/GO foams could completely remove MB by taking half the time. The adsorptions of MB on both ROM and ROM/GO foams could reach equilibrium within 10 min and were interpreted well by a pseudo-first-order kinetic model with high correlation coefficient (*R*^2^) values (Figure 2c). The results manifest that coating the synthesized GO on the ROM foam surfaces not only enhances the adsorption performance of the foams but also accelerates the reduction rate six times higher than that of the foams.

### 3.3 Reusable Evaluation of ROM/GO Foams

**Figure 3:**
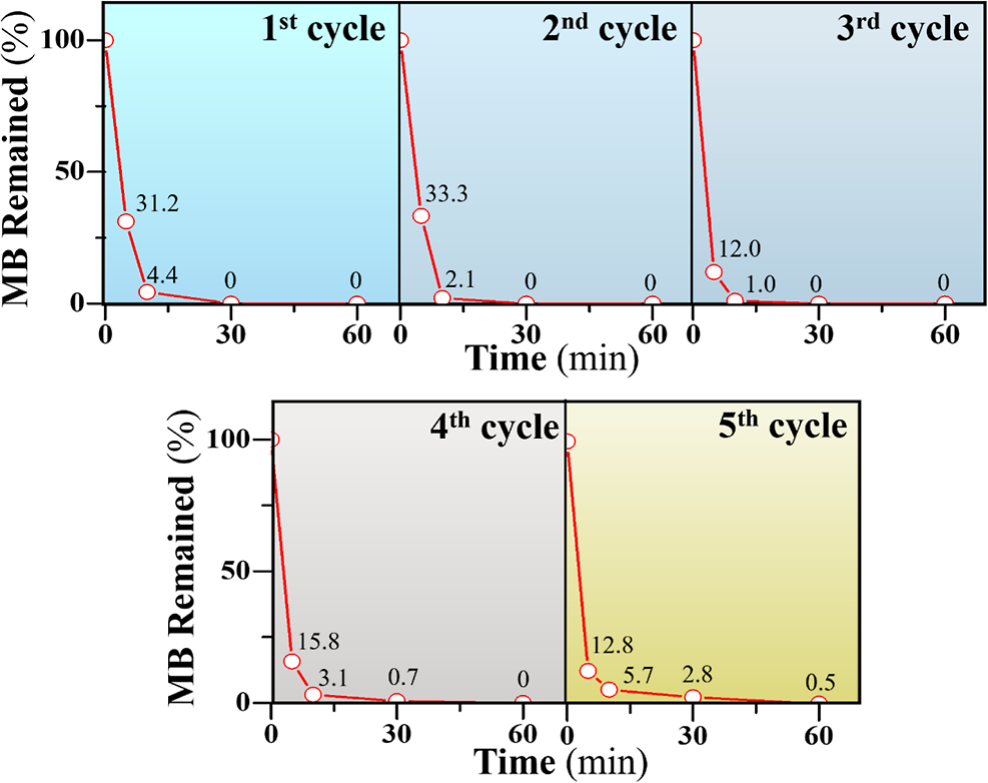
Effectively reusable profiles of the ROM/GO foams.

The ability to reuse the adsorbents is more significant when applying the adsorbents for practical applications. The adequate adsorbents should be simply reused multiple times without a noticeable decline in their performance in order to make the treatment process economically and environmentally viable. Nevertheless, the GO layer has been reported to be less stable in water because of its hydrophilic property.^36^ As carefully observed, neither scraps of the ROM foams nor the synthesized GO were released from the coated layers during the desorption tests. It indicates the stability of GO layers, which may be relevant to the impurities in the synthesized GO derived from the graphite pencil leads. Figure 3 states that the adsorption activity of the ROM/GO foams can remain nearly 100% even after 5 cycles of reuse. A common issue with reusing GO is that the powder form of GO is reportedly difficult to separate after adsorption. A mass loss can occur upon recycling, leading to a decrease in recycling efficiency.^3^ Notably, no mass loss was found in the ROM/GO foams during the tests. According to their superior readsorption efficiency, the ROM/GO foams can reduce the cost of operation and simplify the treatment process, making them suitable for large-scale application.

Compared to other technologies applied for MB removal, such as photocatalytic technology, the ROM/GO foams could provide similar removal efficiency, using immobilized TiO_2_ on the cork substrate (98.96%).^37^ However, the removal performance of the latter was slightly decreased to 78.13% after five cycles. Although photocatalytic technology is a promising approach to degrade water pollutants, its performance relies on light irradiation, resulting in a limitation of reactors designed properly for practical application. The high adsorption ability of the ROM/GO foams was described by the combined properties of the ROM foams and the coated GO. The MB adsorption efficiencies of different materials are shown in Table 1. A thorough search of the relevant literature revealed no relevant research on MB removal using porous ceramic supports coated with GO. However, few studies reported the use of open-cell polymeric supports such as sponges.^30,31^ It indicates that the ROM/GO can compete with those GO-composited sponge foams and GO-composited membranes.^32^

**Table 1:** Comparison of Adsorption Efficiencies of Different Support Materials Coated by GO

samples	initial concentrations of MB (ppm)	adsorption times (min)	adsorption efficiencies (%)	*K* (1/min)	testing conditions	references
ROM foams	5	60	51.0	0.049	batch adsorption	this study
ROM/GO foams			100.0	0.312		
sponge/GO composites	10		99.3	0.025	continuous removal	Nayl et al.,^30^ 2019
	30		>90.0			
	50		41.5			
melamine sponge coated GO	50	240	94.9	0.329	batch adsorption	Zhu et al.,^31^ 2023
GO/cellulose acetate membranes	10		99.0		vacuum filtration	Zeng et al.,^32^ 2017

### 3.4 Properties of ROM Foams

**Figure 4:**
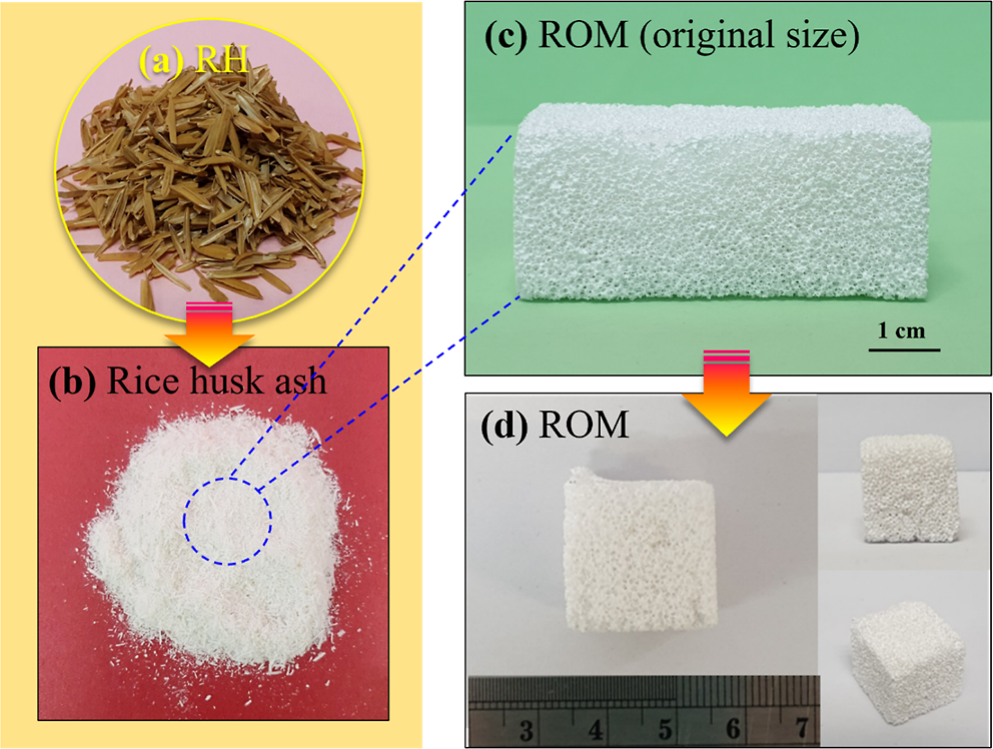
Photographs of (a) as-received RH, (b) calcined RH designated as RHA, (c) ROM/GO foam, and (d) ROM/GO foam after cutting into 2 pieces with the specific size.

**Figure 5:**
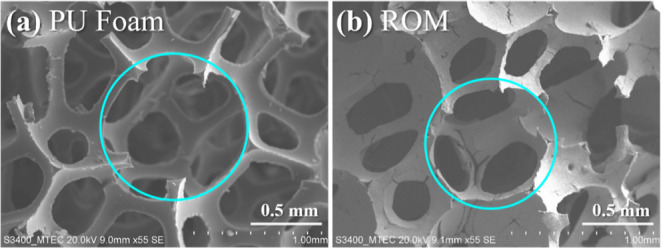
SEM images of (a) as-received PU foam and (b) sintered ROM foam showing pore characteristics.

**Figure 6:**
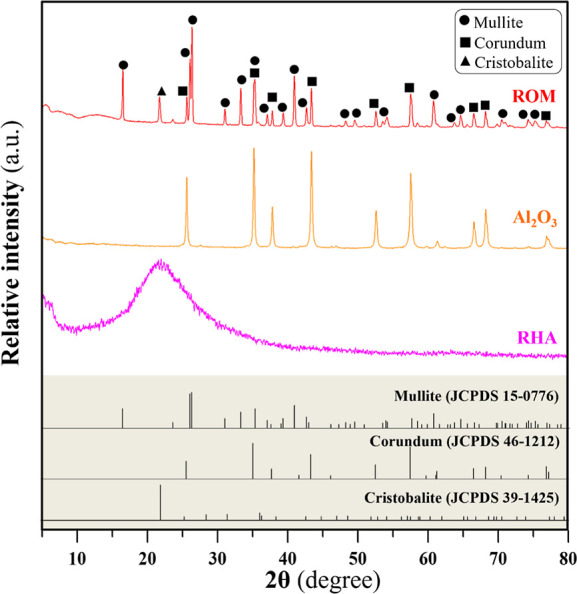
XRD profiles of a ROM foam compared with Al_2_O_3_ and calcined RH designated as RH.

Figure 4a shows the as-received RH compared with calcined RH, designated as RH ash (RHA) as shown in Figure 4b. The ROM foam, as shown in Figure 4c, revealed structural integrity at a length of 60 mm. No cracks were found after cutting into the specific size, as shown in Figure 4d. As compared to the 3D interconnected pore structure of a 45 ppi PU foam (Figure 5a), the ROM foam could maintain the original structure of the PU foam without structural deformation (Figure 5b). The pore sizes of all ROM foams decreased after the sintering process, compared with the PU foams. Figure 6 shows the XRD profile of the ROM foam compared with the profiles of Al_2_O_3_ and RHA. The foam was composed of mullite as the major component, with minor phases of corundum and cristobalite.

Considering the physical and mechanical properties of the ROM foams as presented in Table 2, the foams possess high porosity with an average of 89%, which is advantageous for the adsorption of contaminants as well as the GO attachment during the coating process. The appropriate mechanical properties of the ROM foams suggest that the foams are suitable for use as ceramic supports.

**Table 2:** Physical and Mechanical Properties of the ROM Foams

properties	ROM foams
phase contents (%)	mullite 70.2/corundum 25.8/cristobalite 4.0
bulk density (g/cm^3^)	0.29
porosity (%)	89
pore size (μm)	cell size 773/window size 278
average flexural strength (MPa)	0.46
average flexural modulus (MPa)	10.74

### 3.5 GO Characteristics

**Figure 7:**
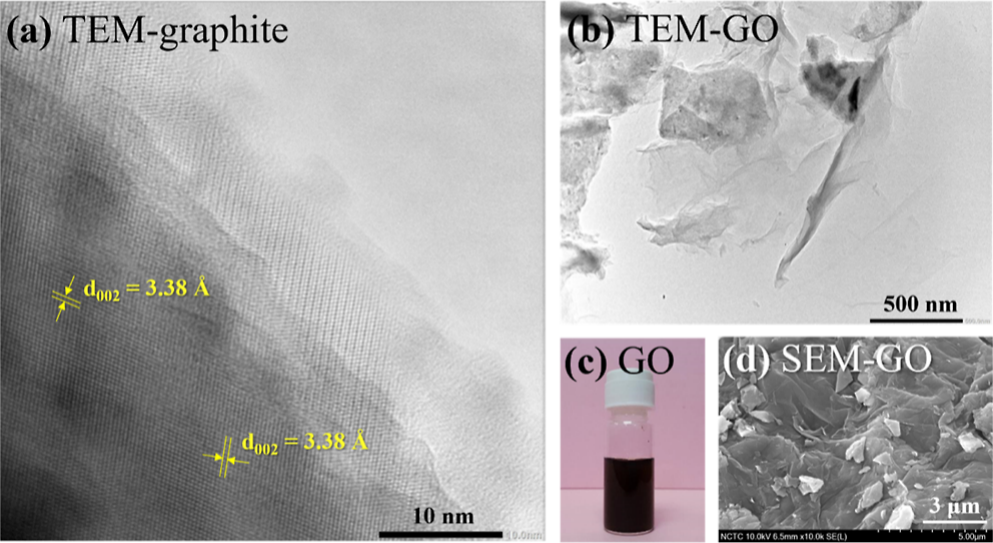
(a) HR-TEM image of graphite pencil leads, (b) bright field TEM image of GO, (c) GO in aqueous solution, and (d) SEM image observed on the surface of GO.

Apart from the structure of the ROM foams, the unique physicochemical properties of the synthesized GO are one of the pivotal factors affecting the ROM/GO foam performance. The high-resolution TEM image taken for graphite pencil leads in Figure 7a shows a lattice fringe corresponding to a graphitic material with a typical interlayer distance of 3.38 Å. Figure 7b shows the morphological structure of GO observed by TEM, exhibiting transparent GO sheets with folded edges. GO could be fully suspended in water (Figure 7c). A high surface area was believed to be the result of the 2D sheets observed in the figure, which was beneficial to improving the adsorption efficiency of the ROM/GO foams. Figure 7d displays a SEM image of dried GO with a conjugated ribbon morphology and wrinkles.

**Figure 8:**
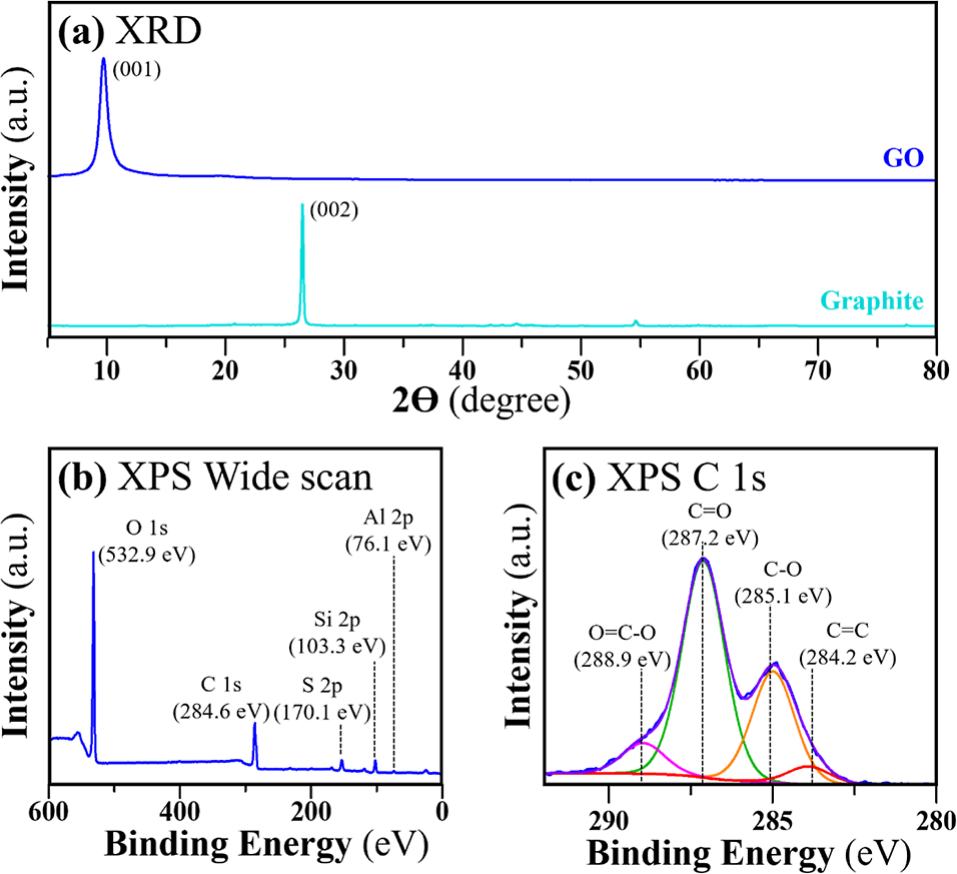
(a) XRD profiles of GO compared with the graphite pencil leads and (b) XPS wide scan spectrum of GO and (c) deconvoluted C 1s spectra of GO.

The successful preparation of GO was further confirmed by the XRD profile shown in Figure 8a. The typical (001) crystal plane of GO was found at 8.80° 2θ. The interplanar distance was calculated to be 1.00 nm, which was larger than that of the original graphite (0.34 nm). It evidences the successful oxidation of graphite to GO. The C and O elements are mainly observed in the XPS survey spectra of GO at 284.6 and 532.6 eV, respectively, as depicted in Figure 8b. The peak at 170.1 eV assigned to the S 2p peak is ascribed to the remaining H_2_SO_4_ impurities. The small peaks corresponding to Si 2p and Al 2p are found at 103.3 and 76.1 eV, possibly originating from the clay binder used in the production of graphite pencil leads.^38^ The presence of these contaminants could be cross-linked with the GO layers, increasing the interfacial adhesion between each layer of GO, resulting in stabilizing the GO layers.^7^ It made the GO layers remain intact, with the foams even performing under turbulent water several times. The identification of oxygen-containing functional groups on the surface of the GO powder, including aromatic ring (C–C/C=C), epoxy and hydroxyl (C–O), carbonyl (C=O), and carboxyl groups (O=C–O), was confirmed by the deconvoluted C 1s spectra (Figure 8c). These oxygenated functional groups on the GO surface would facilitate the adsorption performance of the material. Since these functional groups are negatively charged, the cationic dye molecules of MB can easily be adsorbed on the surfaces of the ROM/GO foams by electrostatic interaction and π–π conjugating interaction.

### 3.6 Proposed Mechanism for MB Adsorption

The comprehensive characterization shows that our ROM/GO foams are fully covered by layers of GO, which is evident from the uniform black color. We anticipate that during the dip-coating process, GO sheets come into contact with the ROM foams. The GO sheets are interconnected to each other and form thin layers on the foam surfaces. The stable GO coating can be attributed to the hydrogen bonding as well as the strong interfacial adhesion between each layer of GO, as explained previously. While the adsorption results indicate that MB was partially adsorbed by the bare ROM foams. In the case of ROM/GO foams, the adsorption of MB by the mullite structure in ROM foams is negligible due to the micropore structure of the foams being covered with GO. Therefore, mechanisms for MB removal by the ROM/GO foams are mainly driven by the surface adsorption of GO. This process is involved by (i) electrostatic attraction between the negatively charged GO layer and positively charged MB molecules, (ii) π–π stacking interaction between the aromatic rings of MB and GO, and (iii) hydrogen bonding between nitrogen atoms of MB and hydrogen atoms of GO. These mechanisms are also supported by the XPS results, which demonstrated the presence of oxygen functional groups on the surface of GO. Furthermore, the interconnected porous structure of the ROM/GO foams is remarkably advantageous for MB removal. It increases the opportunity for MB molecules to contact the macrostructure of the ROM/GO foams. This unique structure facilitates the diffusion of MB into the framework structure, with abundant adsorption sites through the boundary layers to the GO surfaces. Consequently, the adsorption performance of the foams was further enhanced.

## 4 Conclusions

The ROM/GO foams, performing as an effective adsorbent for MB removal, were fabricated via the facile and scalable method of the replica technique. GO was successfully coated on the surfaces of the ROM foams by using a simple dip-coating technique. The experimental results revealed that the ROM/GO foams can completely remove (100%) MB from the aqueous solution within 30 min. The reusability studies confirmed that the ROM/GO foams had the excellent property of repeatable use for more than 5 cycles without a significant decline in the adsorption capacity. The results obtained from this study also suggested that the adsorption mechanism of the ROM/GO foams toward MB removal was possibly dominated by electrostatic interaction and π–π interaction. When considering a separation process with a rapid and simple approach after using the absorbent material, the ROM/GO foams can be applied in futuristic wastewater treatment technology as sustainable adsorbent foams, having effective functionality for the removal of dye contamination. This concept study could be extended to other pollutants of emerging concern in further study.
